# Supplementary material for: An open-source framework for large-scale, flexible evaluation of biomedical text mining systems
Source: J Biomed Discov Collab. 2008 Jan 29;3:1. doi: 10.1186/1747-5333-3-1 (PMC2276192; doi:10.1186/1747-5333-3-1)
Supplement: Additional file 1 — Evaluation platform source code. The additional file contains the software evaluation framework discussed in this paper. The most current release can be downloaded from SourceForge.net [1]. See the accompanying README file for instructions on installation and use. Also included in the distribution is a collection of UIMA wrappers for some commonly used BioNLP tools and annotated corpora. [file 1747-5333-3-1-S1.zip › bionlp_uima_repository-1.0.1/docs/api/src-html/edu/uchsc/ccp/uima/ae/util/filter/SlotRemovalFilter_AE.html]

```
001    package edu.uchsc.ccp.uima.ae.util.filter;
002    
003    import java.util.ArrayList;
004    import java.util.HashSet;
005    import java.util.Iterator;
006    import java.util.List;
007    import java.util.Set;
008    
009    import org.apache.uima.analysis_engine.ResultSpecification;
010    import org.apache.uima.analysis_engine.annotator.AnnotatorConfigurationException;
011    import org.apache.uima.analysis_engine.annotator.AnnotatorContext;
012    import org.apache.uima.analysis_engine.annotator.AnnotatorContextException;
013    import org.apache.uima.analysis_engine.annotator.AnnotatorInitializationException;
014    import org.apache.uima.analysis_engine.annotator.AnnotatorProcessException;
015    import org.apache.uima.analysis_engine.annotator.JTextAnnotator_ImplBase;
016    import org.apache.uima.jcas.JCas;
017    import org.apache.uima.jcas.cas.FSArray;
018    
019    import edu.uchsc.ccp.uima.annotation.CCPDocumentInformation;
020    import edu.uchsc.ccp.uima.annotation.CCPTextAnnotation;
021    import edu.uchsc.ccp.uima.mention.CCPClassMention;
022    import edu.uchsc.ccp.uima.mention.CCPSlotMention;
023    
024    /**
025     * removes slots from a specific class mention. This class is a general utility class first created to remove unnecessary slots from a gold standard
026     * set so that the AnnotationComparator would be able to compare only the desired slots among annotations.<br>
027     * <br>
028     * Input must be a pipe-delimited class name | slot name pairing<br>
029     * e.g. an entry of protein|entrez_gene_id would remove all entrez_gene_id slots from any protein mention
030     * 
031     * @author williamb
032     * 
033     */
034    public class SlotRemovalFilter_AE extends JTextAnnotator_ImplBase {
035    
036        private final boolean DEBUG = false;
037    
038        public static final String PARAM_SLOTS_TO_REMOVE_LIST = "SlotsToRemoveList";
039    
040        private Set<String> slotsToRemoveList;
041    
042        private Set<String> classesOfInterest;
043    
044        private boolean REMOVE_ALL_SLOTS = false;
045    
046        @Override
047        public void initialize(AnnotatorContext ac) throws AnnotatorInitializationException, AnnotatorConfigurationException {
048            /* read in input parameters, and initialize a list of slots to remove */
049            try {
050                String[] classMentionSlotMentionPairsToRemove = (String[]) ac.getConfigParameterValue(PARAM_SLOTS_TO_REMOVE_LIST);
051                slotsToRemoveList = new HashSet<String>();
052                classesOfInterest = new HashSet<String>();
053                for (String pairing : classMentionSlotMentionPairsToRemove) {
054                    if (pairing.equalsIgnoreCase("ALL")) {
055                        REMOVE_ALL_SLOTS = true;
056                    }
057                    slotsToRemoveList.add(pairing.toLowerCase());
058                    try {
059                        String className = pairing.substring(0, pairing.indexOf("|")).toLowerCase();
060                        classesOfInterest.add(className);
061                        debug("Adding class of interest: " + className);
062                    } catch (StringIndexOutOfBoundsException siobe) {
063                        error("Incorrect format for adding a slot to be removed, make sure the format is className|slotName: " + pairing);
064                    }
065                }
066            } catch (AnnotatorContextException e) {
067                e.printStackTrace();
068            }
069            super.initialize(ac);
070        }
071    
072        /**
073         * cycle through all annotations and remove any slots that have been inputted by the user
074         */
075        public void process(JCas jcas, ResultSpecification arg1) throws AnnotatorProcessException {
076    
077            List<CCPTextAnnotation> annotationsToRemove = new ArrayList<CCPTextAnnotation>();
078    
079            Iterator annotIter = jcas.getJFSIndexRepository().getAnnotationIndex(CCPTextAnnotation.type).iterator();
080    
081            
082            /* get document ID */
083            Iterator docInfoIter = jcas.getJFSIndexRepository().getAnnotationIndex(CCPDocumentInformation.type).iterator();
084            String documentID = "-1";
085            if (docInfoIter.hasNext()) {
086                CCPDocumentInformation srcDocInfo = (CCPDocumentInformation) docInfoIter.next();
087                documentID = srcDocInfo.getDocumentID();
088            }
089            
090            System.err.println("Removing slots for: " + documentID);
091            
092            
093            while (annotIter.hasNext()) {
094                Object possibleAnnot = annotIter.next();
095                if (possibleAnnot instanceof CCPTextAnnotation) {
096    
097                    CCPTextAnnotation ccpTA = (CCPTextAnnotation) possibleAnnot;
098                    CCPClassMention ccpCM = ccpTA.getClassMention();
099    
100                    if (REMOVE_ALL_SLOTS) {
101                        FSArray ccpSlots = new FSArray(jcas, 0);
102                        ccpCM.setSlotMentions(ccpSlots);
103                    } else {
104    
105                        String mentionName = ccpCM.getMentionName().toLowerCase();
106                        debug("MentionName: " + mentionName + "  MENTION NAMES OF INTEREST: " + classesOfInterest.toString());
107                        if (classesOfInterest.contains(mentionName)) {
108                            debug("Found class of interest: " + mentionName);
109    
110                            FSArray ccpSlots = ccpCM.getSlotMentions();
111    
112                            /*
113                             * since the FSArray class has no remove() method, we will create a set of CCPSlotMentions that we will keep. If any slots are
114                             * removed, they will not be added to this list, and then this new list will be put into a new FSArray, and replace the
115                             * original FSArray. This is a bit of a work-around... perhaps there's a better way.
116                             */
117                            List<CCPSlotMention> slotsToKeep = new ArrayList<CCPSlotMention>();
118                            boolean removedAtLeastOneSlot = false;
119                            for (int i = 0; i < ccpSlots.size(); i++) {
120                                CCPSlotMention ccpSM = (CCPSlotMention) ccpSlots.get(i);
121                                String slotName = ccpSM.getMentionName().toLowerCase();
122                                if (slotsToRemoveList.contains(mentionName + "|" + slotName)) {
123                                    debug("Found slot of interest: " + slotName);
124                                    /* then remove this slot */
125                                    removedAtLeastOneSlot = true;
126                                } else {
127                                    /* we are not going to remove this slot, so we store it in the list */
128                                    slotsToKeep.add(ccpSM);
129                                }
130                            }
131    
132                            /* if we removed a slot, then we need to replace the FSArray for this CCPClassMention */
133                            if (removedAtLeastOneSlot) {
134                                FSArray keptSlots = new FSArray(jcas, slotsToKeep.size());
135                                for (int i = 0; i < keptSlots.size(); i++) {
136                                    keptSlots.set(i, slotsToKeep.get(i));
137                                }
138                                ccpCM.setSlotMentions(keptSlots);
139                            }
140                        }
141                    }
142    
143                } else {
144                    System.err.println("WARNING -- AnnotatorFilter_AE: CCPTextAnnotation expected but instead got "
145                            + possibleAnnot.getClass().getName());
146                }
147            }
148    
149            /* now remove annotations that had class mention types not in the classMentionTypesToKeep list */
150            for (CCPTextAnnotation ccpTA : annotationsToRemove) {
151                ccpTA.removeFromIndexes();
152                ccpTA = null;
153            }
154    
155        }
156    
157        private void error(String message) {
158            System.err.println("ERROR -- SlotRemovalFilter: " + message);
159        }
160    
161        private void debug(String message) {
162            if (DEBUG) {
163                System.err.println("DEBUG -- SlotRemovalFilter: " + message);
164            }
165        }
166    
167    }
```
